# A new FRDA mouse model [*Fxn*^null^:YG8s(GAA) > 800] with more than 800 GAA repeats

**DOI:** 10.3389/fnins.2023.930422

**Published:** 2023-01-26

**Authors:** Ester Kalef-Ezra, Fred Jonathan Edzeamey, Adamo Valle, Hassan Khonsari, Paula Kleine, Carlo Oggianu, Sahar Al-Mahdawi, Mark A. Pook, Sara Anjomani Virmouni

**Affiliations:** ^1^Ataxia Research Group, Division of Biosciences, Department of Life Sciences, Brunel University London, Uxbridge, United Kingdom; ^2^Energy Metabolism and Nutrition, Research Institute of Health Sciences (IUNICS), University of Balearic Islands, Palma, Spain; ^3^Health Research Institute of Balearic Islands (IdISBa), Palma, Spain; ^4^Biomedical Research Networking Center for Physiopathology of Obesity and Nutrition (CIBERobn CB06/03/0043), Instituto de Salud Carlos III, Madrid, Spain

**Keywords:** Friedreich’s ataxia, FRDA, *FXN*, frataxin, GAA repeat, mouse model, YG8JR, Y47JR

## Abstract

**Introduction:**

Friedreich’s ataxia (FRDA) is an inherited recessive neurodegenerative disorder caused by a homozygous guanine-adenine-adenine (GAA) repeat expansion within intron 1 of the *FXN* gene, which encodes the essential mitochondrial protein frataxin. There is still no effective therapy for FRDA, therefore the development of optimal cell and animal models of the disease is one of the priorities for preclinical therapeutic testing.

**Methods:**

We obtained the latest FRDA humanized mouse model that was generated on the basis of our previous YG8sR, by Jackson laboratory [YG8JR, *Fxn*^null^:YG8s(GAA) > 800]. We characterized the behavioral, cellular, molecular and epigenetics properties of the YG8JR model, which has the largest GAA repeat sizes compared to all the current FRDA mouse models.

**Results:**

We found statistically significant behavioral deficits, together with reduced levels of frataxin mRNA and protein, and aconitase activity in YG8JR mice compared with control Y47JR mice. YG8JR mice exhibit intergenerational GAA repeat instability by the analysis of parent and offspring tissue samples. Somatic GAA repeat instability was also detected in individual brain and cerebellum tissue samples. In addition, increased DNA methylation of CpG U13 was identified in *FXN* GAA repeat region in the brain, cerebellum, and heart tissues. Furthermore, we show decreased histone H3K9 acetylation and increased H3K9 methylation of YG8JR cerebellum tissues within the *FXN* gene, upstream and downstream of the GAA repeat region compared to Y47JR controls.

**Discussion:**

These studies provide a detailed characterization of the GAA repeat expansion-based YG8JR transgenic mouse models that will help investigations of FRDA disease mechanisms and therapy.

## Introduction

Friedreich’s ataxia (FRDA, ORPHA 95, OMIM 229300) is a multisystemic, mitochondrial disorder with an average age of onset in childhood (5–15 years old) and death of 37 years old (Harding, 1981; Mateo et al., 2003). It is the most common inherited ataxia with an estimated prevalence of 1 in 20,000 to 1 in 50,000 in the Caucasian population (Campuzano et al., 1996). Clinically, it is characterized by progressive spinocerebellar neurodegeneration that affects mainly the dorsal root ganglia (DRG) and cerebellum and it is linked to progressive ataxia of limbs and gait, as well as dysarthria (Delatycki and Corben, 2012). Moreover, FRDA patients have increased incidence of hypertrophic cardiomyopathy, diabetes mellitus, glucose intolerance, blindness, and deafness (Cook and Giunti, 2017).

Approximately 96% of FRDA individuals are homozygous for a guanine-adenine-adenine (GAA) repeat expansion mutation within intron 1 of the frataxin (*FXN*) gene locus (Campuzano et al., 1996). Around 4% of affected individuals are having a GAA expansion in one allele and a different pathogenic mutation in the other allele of the *FXN* gene (Campuzano et al., 1996). Healthy individuals have 5–40 GAA repeat triplets, however, FRDA affected individuals exhibit larger expansions ranging from 70 to more than 1700 GAA repeats. These mutations cause *FXN* gene silencing resulting in 4 to 29% of protein expression levels in FRDA patients compared to the normal individuals (Campuzano et al., 1997). The precise mechanism of the partial *FXN* reduction in relation to the extended GAA repeats is still not fully understood, but it is suggested to be caused due to the formation of (a) heterochromatin (Greene et al., 2007), (b) sticky DNA (Sakamoto et al., 1999), (c) DNA-RNA hybrids (Grabczyk et al., 2007), and/or (d) frataxin transcriptional repressive environment (Silva et al., 2015). The *FXN* gene encodes the mitochondrial protein frataxin which is essential for the embryonic development. The exact function of frataxin is still controversial, but it has been shown that it plays a key role in iron-sulfur cluster (ISC) protein and heme biosynthesis (Gerber et al., 2003). Frataxin deficiency has been linked to increased oxidative stress (Wong et al., 1999), induced sensitivity to ionizing radiation (Chamberlain and Lewis, 1982; Khonsari et al., 2016) and impairments on DNA repair pathways (Chamberlain and Lewis, 1982). Additionally, decrease in frataxin levels has been associated with increased telomere shortening (Castaldo et al., 2013; Anjomani Virmouni et al., 2015a), deficiencies in mitochondrial respiratory complexes I, II, and III (Rötig et al., 1997), and accumulation of mitochondrial iron (Babcock et al., 1997; Delatycki et al., 1999). Recently, it was suggested that frataxin has a potential role in lipoprotein peroxidation (Navarro et al., 2010; Abeti et al., 2016).

At present, there is no effective therapy to reverse or delay the progression of the disease (Tai et al., 2018), suggesting the need for better cell and animal FRDA models to increase preclinical therapeutic testing. So far, various cell and animal models have been used to study the FRDA pathogenesis from yeast to mammals (Lupoli et al., 2018). However, none of them represents all key complex features of FRDA phenotype, but only part of them. Human and mouse frataxin share 73% identity (Koutnikova et al., 1997), suggesting that mice could serve as good FRDA animal models. We have previously shown that human *FXN* can replace mouse *Fxn* by generating YAC transgenic mice expressing human *FXN* having normal GAA repeats (9 GAA repeats) on a mouse *Fxn* null background (deletion on exon 4 of *Fxn*), designated as Y47R (Pook et al., 2001) (available from The Jackson Laboratory, ME, United States as stock number 024097). We have also generated FRDA YAC transgenic mouse models containing a large human *FXN* genomic transgene with GAA repeats, designated as YG8 (190 and 120 repeats) and YG22 (190 GAA repeats), as well as the “rescued” mice, YG8R and YG22R (Al-Mahdawi et al., 2004, 2006, 2008; Anjomani Virmouni et al., 2014; Sandi et al., 2014). By employing selective breeding, we have produced YG8-derived FRDA mice lines with GAA repeat expansion sizes of approximately ∼300 repeats (YG8sR, available from The Jackson Laboratory, ME, United States stock number 024113) (Anjomani Virmouni et al., 2015b). These mice exhibit significantly decreased levels of frataxin expression in all tested tissues and exhibited a rather mild, late-onset progressive FRDA phenotype.

Generating mouse models with high GAA repeats is crucial to study FRDA pathogenesis, since most patients have more than 300 GAA repeat expansions (Campuzano et al., 1996) and there is an inverse correlation between the number of GAA repeats in the smaller allele with the disease severity and the age of onset of the disease (Filla et al., 1996). Here, we characterized the latest FRDA humanized model containing a larger expansion of more than 800 GAA repeats, designated as YG8JR [*Fxn*^null^:YG8s(GAA) > 800]. The YG8JR mice, which originated from our previous YG8sR mice, were compared with the humanized control mice designated Y47JR [*Fxn^em2⋅1Lutzy^* Tg(FXN)Y47Pook/J], containing a normal number of GAA repeats (9 repeats). The YG8JR and Y47JR mice underwent a partially different genetic manipulation of the other YAC FRDA mouse models (YG8R, YG22R, and YG8sR that harbor deletion on exon 4 of *Fxn*, Anjomani Virmouni et al., 2014, 2015a) in which the *Fxn*-knockout was generated by CRISPR/Cas9 and Cre loxP-mediated deletion of exon 2. To our knowledge, this model has the largest GAA repeat size of all the current FRDA mouse models. Here we characterized the behavioral, cellular, and epigenetic features of the YG8JR mice. Overall, we show that YG8JR mice exhibit decreased motor coordination and locomotor activities, reduced frataxin expression, impaired ISC protein synthesis, and epigenetic silencing of *FXN* which, altogether, are in line with FRDA-like signature.

## Materials and methods

### Animal procedures

The founder YG8JR [*Fxn^em2⋅1Lutzy^* Tg(FXN)YG8Pook/800J, stock no: 030395, RRID:IMSR_JAX:030395 and Y47JR [*Fxn^em2⋅1Lutzy^* Tg(FXN)Y47Pook/J, stock no: 031007, RRID:IMSR_JAX:031007 mice were obtained from The Jackson Laboratory, ME, United States. The Y47R control mice (Pook et al., 2001) used in this study are also available from The Jackson Laboratory, ME, United States (stock no: 024097, RRID:IMSR_JAX:024097). Mice were housed in conventional open cages with Litaspen Premium 8/20 bedding, paper wool nesting and standard fun tunnel environmental enrichment. The animal husbandry was carried out at 11 h dark versus 13 h light, 20–23°C, and 45–60% humidity. The mice were nourished with a diet of SDS RM3 expanded food pellets and standard drinking water. The animal study was reviewed and approved by Brunel University Animals Welfare and Ethical Review Board and all procedures were carried out in accordance with the UK Home Office “Animals (Scientific Procedures) Act 1986.”

### Behavioral testing

Weighing, Rotarod, Beam-walk, and Beam-breaker tests were performed as previously described (Anjomani Virmouni et al., 2015b). Motor coordination ability was assessed using a Ugo-Basille 7650 accelerating rotarod treadmill. Four trials were performed with the speed of the rotation gradually increasing from 4 to 40 rpm and each trial lasted approximately 3–5 min, separated by a rest period of 200 s between each trial. The time taken for the mouse to fall from the apparatus was recorded and the maximum time on the rotarod was set at 400 s. Beam-breaker locomotor activity test (Average velocity, ambulatory distance, vertical counts, vertical time, and jump counts) were measured over a 1-min period and repeated five times for each mouse using a beam-breaker activity monitor (MEDOFA-510 activity chamber; Med Associates). The Beam-walk test was carried out using 90-cm long, 12-mm and 22-mm diameter, horizontal wooden beams. Coordination ability was assessed by measuring the time taken for the mouse to cross the beam. Mice received two trainings and were assessed four times on each beam with a rest period of 5 min between each trial. Five mice were used per genotype and were assessed at two different time points (3–4 and 4–5 months old). A total of 10 mice (five males and five females per group) were used for Rotarod and weight analysis of the mice at age of 7–9 months. Data analysis and manipulation was performed using Microsoft Excel.

### GAA repeat analysis

Genomic DNA was extracted from mouse tissues by standard phenol/chloroform extraction and ethanol precipitation. GAA PCR amplification was carried out using GAA-B Forward 5′-AATGGATTTCCTGGCAGGACGC-3′ and GAA-B Reverse 5′-GCATTGGGCGATCTTGGCTTAA-3′ primers, followed by sizing on agarose gels, as previously described (Holloway et al., 2011). GAA repeat sizes were assessed by subtracting 285 bp of flanking non-repeat DNA from the PCR product size and dividing the remaining base pair size by 3.

### Quantitative reverse transcriptase PCR

Total RNA was extracted from YG8JR and Y47JR mouse brain tissues (*n* = 3 each group) by homogenization with Trizol (Invitrogen, MA, United States) and cDNA was then prepared by using QuantiTect Reverse Transcription Kit (Qiagen, Hilden, Germany) following the manufacturer’s instructions. Levels of human transgenic *FXN* mRNA were assessed by qPCR using QuantStudio 7 Flex Real-Time PCR (Applied Biosystems, MA, United States) system and Power SYBR™ Green (Applied Biosystems, MA, United States) with the following primers that equally amplify human and mouse sequences: FRT-I Forward 5′-TTGAAGACCTTGCAGACAAG-3′ and RRT-II Reverse 5′-AGCCAGATTTGCTTGTTTGG-3′, 121-bp amplicon size. Mouse *HPRT* RT-PCR primers used for normalization were as follows: HPRT Forward 5′-ATGAAGGAGATGGGAGGCCA-3′ and HPRT Reverse 5′-TCCAGCAGGTCAGCAAAGAA-3′, 80-bp amplicon size. The qRT-PCR reaction was performed in triplicates in a 96-well plate (Applied Biosystems, MA, United States) in at least two independent experiments. Relative quantification values were identified by 2^–ΔΔ*Ct*^ method.

### Immunoblot analysis

Frozen brain tissues were lysed using cell lysis buffer (Cell Signaling Technology, MA, United States) supplemented with 400 μM PMSF protease inhibitor (Cell Signaling Technology, MA, United States) and incubated on ice for 30 min. Lysates were subjected to centrifugation at 12,000 × *g* for 30 min at 4°C and protein concentrations were determined by Pierce BCA assay (Thermo Fisher Scientific, MA, United States). A total of 50 μg of protein lysates were boiled for 10 min and subjected to SDS-PAGE electrophoresis using 4–15% precast gels (Bio-Rad). Densitometry was calculated using the Image Lab Software 5.2.1 (Bio-Rad Laboratories, CA, United States). Antibodies used in this study are as follows: anti-Frataxin (1:250) (Abcam Cat# ab113691, RRID:AB_10862125), anti-Tubulin (1:10,000) (Abcam Cat# ab6160, RRID:AB_305328), rabbit anti-Rat HRP (1:5000) (Abcam Cat# ab6734, RRID:AB_955450), and goat anti-Mouse HRP (1:300) (Agilent Cat# P0447, RRID:AB_2617137). Non-relevant gel lanes and unspecific bands were excised by digital treatment using Power Point. Original uncropped images are presented as Supplementary material.

### Chromatin immunoprecipitation

ChIP analysis of YG8JR and Y47JR mouse tissues (*n* = 4) was carried out by initial cross-linking of DNA and protein by formaldehyde treatment of homogenized cerebellum tissue samples (45 mg each). DNA was then sheared by sonication using a Bioruptor Pico sonication system (Diagenode), followed by immunoprecipitation with trimethyl-Histone H3 (Lys9) (Millipore Cat# 07-442, RRID:AB_310620) or acetyl-Histone H3 (Lys9) (Millipore Cat# 07-352, RRID:AB_310544) antibodies. The DNA was reverse-cross linked and extracted by standard phenol/chloroform/glycogen extraction and ethanol precipitation. Quantitative PCR amplification of the co-immunoprecipitated DNA was carried out with Fast SYBR Green in a QuantStudio 7 Flex Real-Time PCR (Applied Biosystems, MA, United States) using three sets of *FXN* primers (Pro: promoter, Up: upstream, Down: downstream of GAA repeat region) as previously described (Herman et al., 2006; Al-Mahdawi et al., 2008). Each value of immunoprecipitated DNA was processed in triplicate qPCR analysis. Relative quantification values were identified by 2^–ΔΔ*Ct*^ method and were normalized to the input values. For each experiment, minus antibody immunoprecipitation and ddH_2_O without chromatin samples were used as negative controls.

### MethylScreen analysis

MethylScreen assay was carried out as previously described (Holemon et al., 2007; Al-Mahdawi et al., 2013). The position CpG U13 in the *FXN* upstream GAA repeat region was assayed in brain (*n* = 2), cerebellum (Y47JR *n* = 3, YG8JR *n* = 4) and heart (*n* = 2) tissues from YG8JR FRDA and Y47JR control mice. Genomic DNA was digested with: (1) a methylation-sensitive restriction enzyme (MSRE), (2) a methylation-dependent restriction enzyme (MDRE), (3) both MSRE and MDRE (double digest, DD), and (4) neither MSRE nor MDRE (mock control). The MSREs and MDRE used for CpG 13 were *Eco*72I (Fermentas) and *Mcr*Bc (Fermentas), respectively. Digested DNA was then amplified by quantitative PCR using SYBR Green (Applied Biosystems, MA, United States) in a QuantStudio 7 Flex Real-Time PCR (Applied Biosystems, MA, United States) with the following primers: CpG13 met Forward 5′-GAACCGTCTGGGCAAAGGCCAG-3′ and met Reverse 5′-ATCCCAAAGTTTCTTCAAACACAATG-3′. The qPCR reaction was performed in triplicates in a 96-well plate (Applied Biosystems, MA, United States). Relative quantification values were identified by 2^–ΔΔ*Ct*^ method, where values were calculated as 2^Δ*Ct(mock*–*digest)*^ with the mock value set at 100%. DNA methylation values were then calculated as follows: Densely Methylated (DM) = (MSRE−DD)/(100−DD) × 100; Unmethylated (UM) = (MDRE-DD)/(100-DD) × 100; Intermediately Methylated (IM) = 100−(DM + UM).

### Aconitase assay

Aconitase activities of YG8JR and Y47JR heart and cerebellum tissues (*n* = 8 each group) were determined using the Aconitase assay kit (Cayman Chemical Company, MI, United States) and normalized to citrate synthase activities determined using a Citrate Synthase assay kit (Sigma, MO, United States), as previously described (Sandi et al., 2014; Anjomani Virmouni et al., 2015b). All experiments were performed in triplicates and protein concentrations were determined using Pierce BCA assay (Thermo Fisher). The absorbance detection was carried out using CLARIOstar Microplate Reader (BMG LABTECH, Aylesbury, United Kingdom).

### Statistical analyses

Statistical tests were performed using GraphPad Prism (version 9), the unpaired two-tailed Student’s *t*-test was used to assess the significance of the differences between group data with a significance value set at *p* < 0.05. Normal distribution of the data and homogeneity variance were assessed using D’Agostino & Pearson omnibus/Shapiro–Wilk normality test and *F*-test, respectively.

## Results

### YG8JR mice display behavioral deficits

**FIGURE 1:**
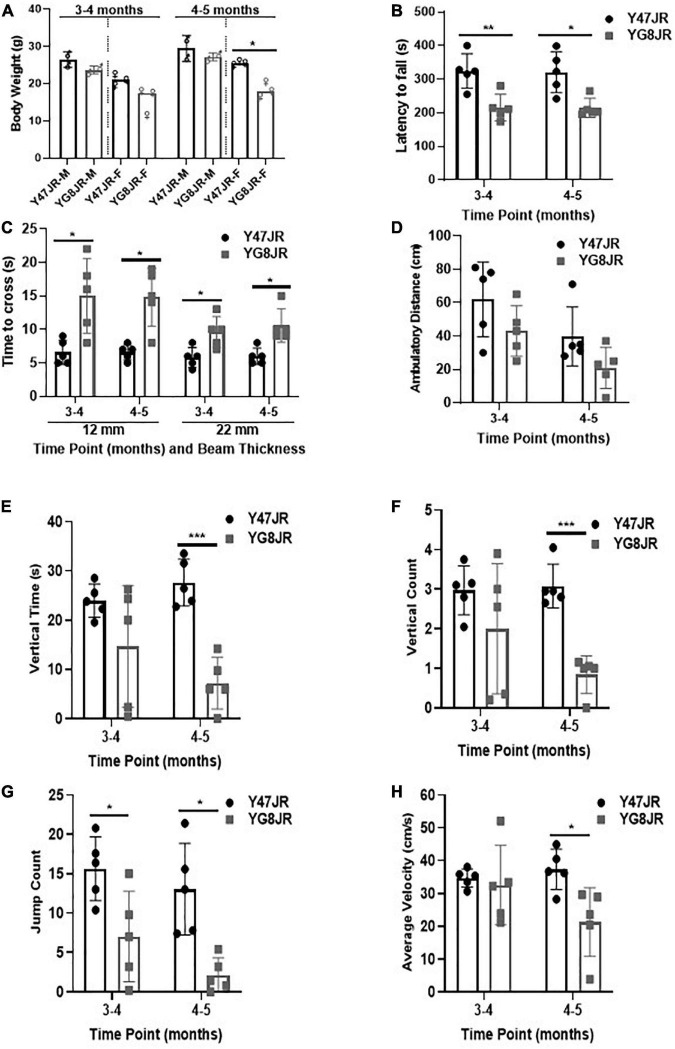
Phenotypic and behavioral analysis of YG8JR FRDA mouse models. **(A)** Body weight (M, male; F, female) and **(B)** Rotarod analysis of YG8JR compared to Y47JR control mice at age 3–4 and 4–5 months old. **(C)** Beam-walk analysis of YG8JR compared to Y47JR control mice at age 3–4 and 4–5 months old using 22 and 12-mm beams. Locomotor activity analysis of YG8JR mice compared with Y47JR control mice assaying. **(D)** Ambulatory distance, **(E)** vertical time, **(F)** vertical count, **(G)** jump count, and **(H)** average velocity. Values represent mean ± SD. Asterisks indicate significant differences between YG8JR and Y47JR, assessed by unpaired two-tailed Student’s *t*-test (*n* = 5 mice per genotype) on time points 3–4 and 4–5 months old (*p < 0.05, **p < 0.01, ***p < 0.001).

We employed various functional assays in order to investigate the behavioral characteristics of YG8JR. The body weight was recorded at two different time points. As shown in Figure 1A, there was no significant difference in the body weight between male YG8JR and Y47JR control mice at both time points, but the female-only values showed statistically significant difference to controls at age of 4–5 months. We also recorded the body weight of YG8JR mice at age of 7–9 months and found a significant decrease in the weight of YG8JR mice compared to Y47JR control mice for both males and females (Supplementary Figures 1A–C). Next, we employed the accelerating rotarod test to investigate the motor coordination and balance of these mice on the same time points. This analysis revealed a statistically significant (3–4 months; *p* < 0.01, 4–5 months: *p* < 0.05) reduction of the duration that the YG8JR mice were able to stay on the accelerating rotarod without falling compared to the Y47JR mice on both time points (Figure 1B and Supplementary Table 1). We also analyzed the motor coordination of the YG8JR mice at age of 7–9 months and found significantly reduced coordination ability of these mice compared to the control Y47JR mice when both males and females were taken together or separately (Supplementary Figures 1D–F). Therefore, we propose that YG8JR exhibit motor incoordination and decreased balance ability. Beam-walk was employed to assess the time needed for YG8JR and Y47JR mice to cross a 12 mm and 22 mm beams. The analysis revealed a statistically significant (*p* < 0.05) delay to transverse both beams for the YG8JR compared to the Y47JR mice at both time points (Figure 1C and Supplementary Table 1), suggesting a decrease in coordination ability of YG8JR compared to the Y47JR control mice.

We tested the locomotor spontaneous activity of YG8JR and Y47JR mice by employing the Beam-breaker test. Five mice were assessed for each group. The YG8JR FRDA mice showed decreased ambulatory distance (total distance covered by each mouse within a specific time) compared to the Y47JR control mice (Figure 1D and Supplementary Table 2). Moreover, the YG8JR mice exhibited a significant decrease in the vertical time (total duration of the mouse standing on hind legs on a specific time) (Figure 1E and Supplementary Table 3) and vertical count (total events of the mouse standing on hind legs on a specific time) compared to the Y47JR control mice at 4–5 months of age (*p* < 0.001) (Figure 1F and Supplementary Table 4). The jump count (total number of the mouse jumps on specific time) of the YG8JR mice was also significantly reduced at both time points (*p* < 0.05) (Figure 1G and Supplementary Table 5). Furthermore, we observed a statistically significant decrease in the average velocity (total distance covered divided by the total time elapsed) of the YG8JR FRDA mice at 4–5 months of age (*p* < 0.05) (Figure 1H and Supplementary Table 6).

### YG8JR mice exhibit somatic and intergenerational GAA repeat instability coupled with reduced frataxin expression levels

**FIGURE 2:**
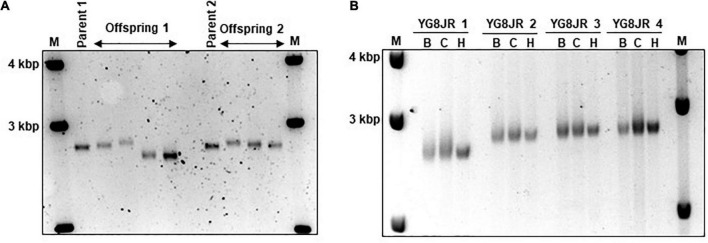
Intergenerational and somatic guanine-adenine-adenine (GAA) repeat analysis. **(A)** Representative example of GelRed stained 1.5% agarose gel determines the GAA PCR sizes of skeletal muscle tissues from parents and offspring YG8JR FRDA mice indicating a range from approximately 820 to 900 GAA repeat units in size within *FXN* gene. Parent 1: male, 7 months old. Parent 2: female, 6 months old. Offspring 1: males, 8–9 months old. Offspring 2: females, 6–9 months old. **(B)** DNA samples from different tissues of YG8JR FRDA mice. YG8JR 1: female, 9 months old. YG8JR 2: male, 9 months old. YG8JR 3: male, 8 months old. YG8JR 4: male, 9 months old. B, brain; C, cerebellum; H, heart. 1 Kb plus DNA ladder was used as the molecular marker (M).

**FIGURE 3:**
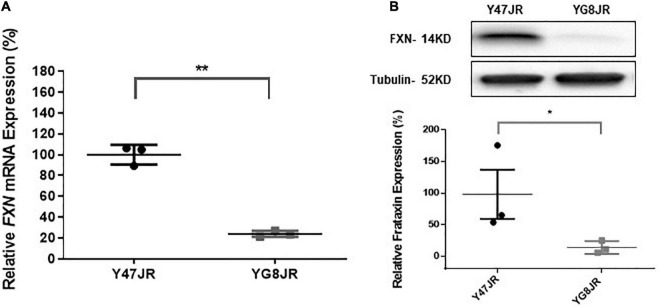
Frataxin expression levels. **(A)** Quantitative RT-PCR analysis of *FXN* mRNA extracted from Y47JR control and YG8JR FRDA mouse brain tissues (*n* = 3 per genotype) using mouse-human specific primers. The experiments were repeated 2–3 times for each genotype and performed in triplicates. Data were normalized to the mean frataxin level of Y47JR samples taken as 100%. Values represent mean ± SD. Asterisks indicate significant differences between YG8JR and Y47JR, assessed by unpaired two-tailed Student’s *t*-test (**p < 0.01). **(B)** Representative Western blot images and relative densitometric bar graphs of Frataxin in brain tissues of YG8JR FRDA and Y47JR control mice (*n* = 3, 2–3 independent experiments). Tubulin was used as protein loading control. Data were normalized to the mean frataxin level of Y47JR samples taken as 100%. Values represent mean ± SD. Asterisks indicate significant differences between YG8JR and Y47JR, assessed by unpaired two-tailed Student’s *t*-test (*p < 0.05).

**FIGURE 4:**
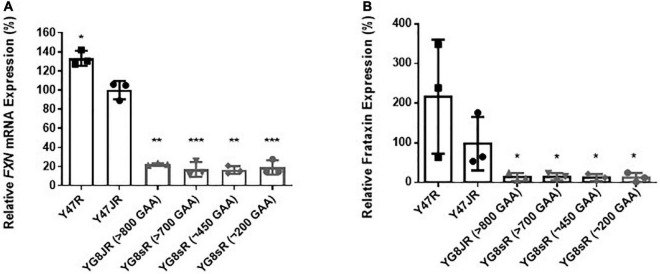
Frataxin expression levels. **(A)** Quantitative RT-PCR analysis of *FXN* mRNA extracted from FRDA and control mouse brain tissues (*n* = 3) using *FXN* mRNA with mouse-human specific primers. The experiments were repeated 2–3 times for each genotype and performed in triplicates. Data were normalized to the mean frataxin level of Y47JR samples taken as 100%. Values represent mean ± SD. Asterisks indicate significant differences compared to Y47JR mice, assessed by unpaired two-tailed Student’s *t*-test (*p < 0.05, **p < 0.01, and ***p < 0.001). **(B)** Relative densitometric bar graphs of Frataxin in brain tissues of FRDA and control mice (*n* = 3, 2–3 independent experiments). Tubulin was used as protein loading control. Data were normalized to the mean frataxin level of Y47JR samples taken as 100%. Values represent mean ± SD. Asterisks indicate significant differences compared to Y47JR control mice, assessed by unpaired two-tailed Student’s *t*-test (*p < 0.05). Two control (Y47R and Y47JR) and four FRDA [YG8JR (>800 GAA), YG8sR (>700 GAA), YG8sR (∼450 GAA), and YG8sR (∼200 GAA)] mice were used.

The GAA repeats are highly dynamic in their nature in FRDA patients, showing both somatic GAA repeat instability throughout life in many tissues, especially in DRG, brain and cerebellum (Montermini et al., 1997) as well as intergenerational GAA repeat instability (De Michele et al., 1998). The expanded GAA length size, particularly that of the shorter one, is inversely correlated with the age of onset of the neurological symptoms, rate of progression, severity, and age of death of FRDA individuals (Long et al., 2017). Indeed, our previous YAC mouse models have shown distinct signs of GAA repeat instability (Al-Mahdawi et al., 2004; Clark et al., 2007; Anjomani Virmouni et al., 2015b). Here, we decided to study the GAA repeat dynamics of YG8JR tissues, due to the different genetic background and longer GAA repeat expansions of this mouse model. Intergenerational instability was assessed between different mouse tissue samples, whereas somatic instability was examined within individual mouse tissue samples. We employed GAA PCR on genomic pooled DNA from different tissue samples and found that the YG8JR mice exhibit intergenerational GAA repeat instability by both maternal and paternal transmissions with approximately 820–900 GAA repeat units in size (Figure 2A and Supplementary Figure 2). Furthermore, YG8JR mice exhibited a smear of expanding GAA repeats in the brain and cerebellum tissues, extending upward (Figure 2B and Supplementary Figure 3). We also determined the *FXN* transcription levels of brain tissues of YG8JR FRDA and Y47JR control mice by quantitative RT-PCR (qPCR). Our data revealed that the YG8JR tissues showed mean values of approximately 24% frataxin mRNA expression compared with the Y47JR control (*p* < 0.01) (Figure 3A). The frataxin protein expression levels were also significantly decreased in the YG8JR mice (21%) compared with the Y47JR control (*p* < 0.05) (Figure 3B and Supplementary Figure 4). Moreover, we compared both protein and mRNA expression levels of frataxin between the YG8JR and the previous YG8sR-derived mouse models with different GAA repeat sizes and there were no significant differences between these mouse models (Figure 4 and Supplementary Figure 5). Nevertheless, the frataxin expression levels were slightly reduced in Y47JR compared to Y47R control mice (Figures 4A, B). Altogether, these data suggest that YG8JR GAA repeat instability and frataxin expression closely resemble *FXN* YAC mouse models with fewer GAA triplet repeats along with those found in human FRDA brain tissues (Al-Mahdawi et al., 2008).

### YG8JR mice show altered epigenetic characteristics

**FIGURE 5:**
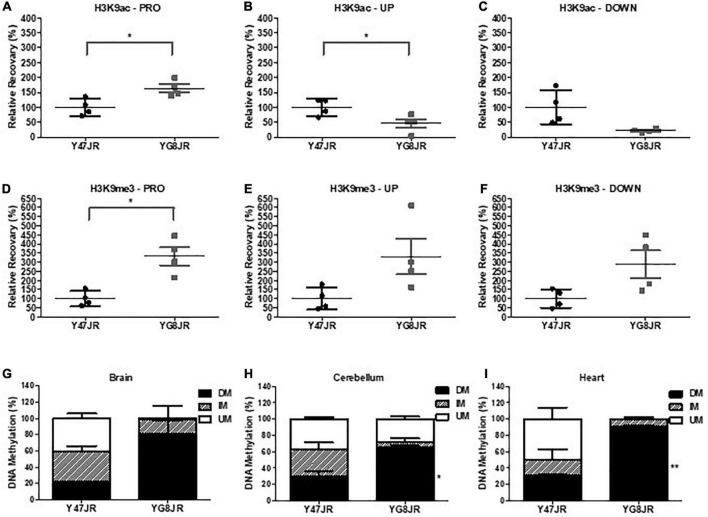
Epigenetic changes within the *FXN* gene and aconitase activity assay. **(A–F)** ChIP quantitative PCR analysis on cerebellum tissues of YG8JR and Y47JR tissues for **(A–C)** acetylated (H3K9ac) and **(D–F)** methylated (H3K9me3) residues within *FXN* **(A,D)** promoter/exon1 (PRO), GAA **(B,E)** upstream (UP), and **(C,F)** downstream (DOWN) region (*n* = 4 mice per group analyzed in triplicates). Results are represented as the relative amount of immunoprecipitated DNA compared with input DNA. YG8JR values were normalized to the Y47JR values that were set as 100%. DNA methylation analysis of CpG U13 in *FXN* GAA repeat region of DNA from YG8JR FRDA and Y47JR control mice in **(G)** brain (*n* = 2 each group), **(H)** cerebellum (Y47JR *n* = 3, YG8JR *n* = 4), and **(I)** heart (*n* = 2 each group) tissues. DM, densely methylated; IM, intermediately methylated; UM, unmethylated. Asterisks indicate significant differences between YG8JR and Y47JR, assessed by unpaired two-tailed Student’s *t*-test (*p < 0.05, **p < 0.01).

Several epigenetic changes have been reported in FRDA cells and tissues (summarized in Sandi et al., 2013). As examples, altered histone modifications have been observed not only on human lymphoblast and fibroblast FRDA cells (Herman et al., 2006; Greene et al., 2007), but also in human and mouse FRDA brain (Al-Mahdawi et al., 2008; Li et al., 2015). Here, we chose to investigate the histone modifications of YG8JR FRDA mouse models in cerebellum, because this tissue plays a key role in FRDA pathology (Koeppen et al., 2007) and frataxin expression is markedly reduced in FRDA cerebellum compared to brain and brain stem tissues (Al-Mahdawi et al., 2006). Moreover, an increased triplet instability has been observed primarily within the cerebellum of both human FRDA patient (De Biase et al., 2007a,b) and transgenic mouse models (Al-Mahdawi et al., 2004; Clark et al., 2007). We carried out chromatin immunoprecipitation (ChIP) analysis from YG8JR and Y47JR cerebellum tissues. The analysis revealed increased acetylation of H3K9 in *FXN* promoter/exon1 region of the YG8JR mice compared to the Y47JR mice (Figure 5A), which was contradictory to the findings in previous YG8R and YG22R mouse models and human FRDA brain tissues (Herman et al., 2006; Al-Mahdawi et al., 2008). However, as with the human samples and previous mouse models, we similarly identified decreased H3K9 acetylation in the upstream and downstream GAA regions of *FXN* in YG8JR mice (Figures 5B, C). Additionally, the data showed an increase in tri-methylated H3K9 throughout all three tested *FXN* regions (Figures 5D–F).

Among epigenetic marks, DNA methylation has been shown to be positively correlated with the GAA triplet length in FRDA patients (Castaldo et al., 2008). Here we employed the MethylScreen analysis to assess the DNA methylation from brain, cerebellum, and heart of YG8JR and Y47JR mice. We chose to investigate the methylation pattern of the CpG U13 in the intron 1 of *FXN* (numbering according to Greene et al., 2007), as this site has one of the highest DNA methylation increase in human FRDA lymphoblastoid lines, cerebellum and heart (Greene et al., 2007; Al-Mahdawi et al., 2013). We observed increased DNA methylation of CpG U13 in *FXN* GAA repeat region on YG8JR compared to Y47JR tissues. More precisely, we observed a non-statistical increase in densely methylated (DM) of 22–80% in brain tissues (Figure 5G). Moreover, we found a statistically significant increase in CpG U13 DM from 30 to 65% (*p* < 0.05) in cerebellum (Figure 5H) and from 30 to 90% (*p* < 0.01) in heart tissues (Figure 5I). These results show a similar pattern compared with those found on cerebellum and heart from human FRDA patients (Al-Mahdawi et al., 2013).

### YG8JR mice reveal reduced aconitase activity

**FIGURE 6:**
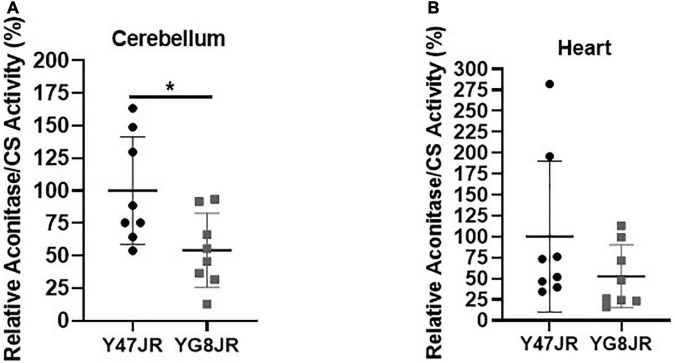
Aconitase activity. Aconitase activity assay on **(A)** cerebellum and **(B)** heart tissues of YG8JR and Y47JR control mice. Relative aconitase activity normalized to citrate synthase (CS) activity from cerebellum and heart tissues (*n* = 8 per group). YG8JR values were normalized to the Y47JR values that were set as 100%. Asterisks indicate significant differences between YG8JR and Y47JR, assessed by unpaired two-tailed Student’s *t*-test (*p < 0.05).

Frataxin insufficiency has been linked to dysregulation of ISC protein cluster synthesis (Gerber et al., 2003) and increased oxidative stress (Wong et al., 1999; Schulz et al., 2000). In order to assess potential effects on ISC cluster synthesis, we measured the aconitase enzyme activities in relation to citrate synthase activities of YG8JR and Y47JR in cerebellum and heart tissue homogenates. YG8JR showed significantly decreased aconitase activity (*p* < 0.05) in the cerebellum tissues (Figure 6A) compared to control mice. No significant difference was observed in the heart tissues of these mice (Figure 6B). These results suggest that YG8JR mice show an imbalanced ISC protein cluster synthesis compared to Y47JR mice.

## Discussion

Friedreich’s ataxia (FRDA) is a rare inherited disorder with variability on the severity of symptoms and the rate of progression among patients. So far, there is no available drug to treat effectively FRDA disease pathogenesis. Therefore, it is crucial to further develop animal and cellular models which reproduce as many human-like FRDA features as possible in order to get insight into the pathogenesis, molecular abnormalities, and therapeutic testing of human FRDA. Various mouse FRDA models have been created by using different technologies, such as the Cre-loxP conditional deletion system, GAA extended repeat sequence knock-in into the mouse *Fxn* intron 1 (reviewed in Perdomini et al., 2013). Furthermore, very recent FRDA models were generated using Tet-On *Fxn*-siRNA (Chandran et al., 2017) or *Fxn-*sgRNA methods (Chen et al., 2016). We have previously generated transgenic mice containing a human *FXN* genomic transgene with normal GAA repeats in a *Fxn* null background, designated as Y47R (Pook et al., 2001). Moreover, we have generated two humanized mouse lines containing extended GAA repeats on a mouse *Fxn* null background, designated as YG8R and YG22R (Al-Mahdawi et al., 2006; Anjomani Virmouni et al., 2014). By natural breeding of the YG8R, we developed a mouse model which has a single copy of the human *FXN* genomic transgene with originally 120 GAA repeat units in size, designated as YG8sR (Anjomani Virmouni et al., 2015b). The YAC FRDA mice represent several key characteristics of cellular FRDA pathogenesis. These mice exhibit not only moderate decrease in frataxin expression, but also mild progressive behavioral deficits, reduced aconitase activities, GAA repeat instability and some epigenetic modifications. These models have been used as powerful tools to understand in depth FRDA pathogenesis (Bourn et al., 2012; Chan et al., 2013; Shan et al., 2013; Hayashi et al., 2014; Chutake et al., 2015; Abeti et al., 2016), on potential novel therapeutic strategies, such as gene replacement therapy (Kemp et al., 2018), gene therapy (Khonsari et al., 2016; Ouellet et al., 2017), as well as in pre-clinical testing of drug compounds (Li et al., 2013; Sahdeo et al., 2014; Abeti et al., 2018; Cherif et al., 2018; Santoro et al., 2018; Mollá et al., 2019). However, these *FXN* YAC transgenic mice show a mild phenotype and none of the currently available models represents all the essential key features observed in human FRDA patients. Hence, it is still critical to generate and characterize animal models with larger GAA repeat extensions that reproduce a more severe FRDA phenotype. Here, we characterized a novel human *FXN* YAC transgenic mouse model that was generated from the YG8sR mouse on a null background with the frataxin floxed on exon 2 allele *Fxn*, made by CRISPR technology by Dr. Cat Lutz at The Jackson Laboratory, ME, United States designated as YG8JR. As a healthy control, we used a humanized mouse model containing the same *Fxn*^null^ allele and a single copy integration of the Y47 human *FXN* YAC transgene, designated as Y47JR. To our knowledge, the YG8JR model is the only humanized FRDA mouse model with the highest GAA repeat number thus far.

A key aspect of YG8JR mice is their motor coordination, locomotor, and balance impairments that are worsened over time. More precisely, YG8JR mice exhibited statistically decreased abilities on rotarod analysis, Beam-breaker and Beam-walk analysis compared to Y47JR mice from 3 to 5 months age. These measurements are in good agreement with the studies on the other YAC FRDA models when compared with Y47R and B6 mice (Sandi et al., 2011; Anjomani Virmouni et al., 2014, 2015b; Mollá et al., 2016). Interestingly, we observed a slightly stronger and earlier onset of these impairments on YG8JR mice compared to the other YAC FRDA models. Moreover, body weight analysis revealed no significant difference between male YG8JR compared to Y47R at age of 3–5 months. The females YG8JR showed statistically reduced body weight compared to the controls at age of 4–5 months. In addition, analysis of the YG8JR mice at age of 7–9 months exhibited significantly reduced body weight compared to controls for both males and females.

Another important characteristic of YG8JR mouse model is that it shows FRDA-like characteristics. Initially, we observed that YG8JR exhibits a drastic reduction of *FXN* mRNA expression of approximately 24% of that of Y47JR control mice, leading to a marked decrease in frataxin protein levels (21%). The change on the *FXN* mRNA and protein expression levels of YG8JR is significantly less than that reported for YG8R and YG22R mice compared to Y47R (Al-Mahdawi et al., 2006, 2008; Anjomani Virmouni et al., 2014) and similar to the values previously reported for YG8sR (Anjomani Virmouni et al., 2015b). These changes of frataxin expression in the YAC mice are in line with studies on FRDA patients which revealed *FXN* mRNA expression of around 23% in brain and 65% in heart tissues (Al-Mahdawi et al., 2008) and protein levels of 4 to 29% compared to the normal individuals (Campuzano et al., 1997). We also compared the expression levels of frataxin between the YG8JR mice and YG8sR-derived mice with different GAA repeats and found no significant difference between these models. However, when we compared the two control models, the levels of frataxin were slightly reduced in the new Y47JR control mice compared to the previous Y47R mice, which may be attributed to their different genetic background. Furthermore, we have previously shown that, of all the FRDA YAC mouse models studied, the greatest frataxin reduction was detected in the YG8sR mice compared to both Y47R human frataxin and B6 mouse frataxin (Anjomani Virmouni et al., 2014, 2015b). It is possible that due to the minimal expression of frataxin in the YG8sR-derived mice, the differences in the expression of frataxin between these lines were not detectable. Secondly, we observed reduced aconitase activities of YG8JR in cerebellum tissues compared of that of Y47JR control mice, likewise previous studies from YG8sR brain tissues (Anjomani Virmouni et al., 2015b).

The YG8JR mice display genetic and epigenetic modifications like those observed in FRDA patients. Our investigations revealed the presence of intergenerational GAA repeat instability analysis of YG8JR tissues by both maternal and paternal transmissions. In the same way, GAA repeat instabilities among generations have been observed in FRDA individuals and in the YAC FRDA models YG8, YG22, YG8R, YG22R, and YG8sR (Al-Mahdawi et al., 2004; Clark et al., 2007; Anjomani Virmouni et al., 2014, 2015b). GAA repeat somatic instability was also observed in the brain and cerebellum tissues of the YG8JR mice. This finding suggests that YG8JR mouse model could be an excellent tool to study the genetic aspects of the disease representing moderate to large GAA repeat expansions. Therefore, the use of these mice is recommended for large-scale studies, to investigate the mechanism(s) of GAA repeat instability and to delineate new therapeutic strategies to achieve triplet contractions or stabilization. On the epigenetics’ level, ChIP analysis revealed that YG8JR mice have increased tri-methylation of H3K9 within *FXN* promoter, upstream and downstream of the GAA repeat region and decreased acetylation of H3K9 within upstream and downstream of the *FXN* GAA repeat region in cerebellum tissues, similarly with previous findings on human lymphoblast cells, fibroblasts, brain tissues, as well as mouse YG22R and YG8R brain tissues (Herman et al., 2006; Al-Mahdawi et al., 2008). While previous studies have shown that the acetylation status of H3K9 within the *FXN* promoter region is decreased in human brain as well as YG8R and YG22R mouse tissues (Al-Mahdawi et al., 2008) or failed to show a clear difference on human FRDA lymphoblast cells (Herman et al., 2006), our data revealed a statistically significant increase in acetylation of H3K9 within the *FXN* promoter region on YG8JR mice compared to Y47JR. These findings are contradictory to expectation from previous studies in YG8R and YG22R mice and human FRDA tissues (Herman et al., 2006; Al-Mahdawi et al., 2008). It is possible that this could be the result of the different tissues tested or due to the differences between the control Y47R and Y47JR mice. Alternatively, it might be due to the different genetic background or the CRISPR methodology used to generate the Y47JR and YG8JR mice. Furthermore, we observed an increase in DNA methylation at the position CpG U13 in the intron 1 of *FXN* gene of YG8JR brain, cerebellum, and heart tissues. These epigenetic alterations are in line with studies on human patients and other FRDA mouse models (Al-Mahdawi et al., 2008, 2013; Castaldo et al., 2008). In heart tissues, the increase in DM of YG8JR was very similar with that observed on FRDA patients (Al-Mahdawi et al., 2013). However, the change in cerebellum tissues of YG8JR mice (DM 30% in control and 65% in FRDA) was not so great as the one observed in human autopsies (DM 14% in control and 96% in FRDA) (Al-Mahdawi et al., 2013). This might be due to the moderate phenotype of the mice or related to the age tested. Overall, these changes suggest that *FXN* gene is highly likely to be under of heterochromatin structure in YG8JR mice.

As a whole, we report that the YG8JR mouse model exhibits progressive FRDA-like pathology with earlier onset, as compared to the previously described *FXN* YAC models with lower GAA repeat expansions. The YG8JR mice can be a useful model for the investigation of FRDA disease mechanisms and therapy.

## Data availability statement

The raw data supporting the conclusions of this article will be made available by the authors, without undue reservation.

## Ethics statement

The animal study was reviewed and approved by Brunel University Animal Welfare and Ethical Review Board.

## Author contributions

SA-M, MP, and SAV conceived and designed the study. EK-E, AV, SA-M, MP, and SAV wrote the manuscript. All authors performed the experiments, read, and approved the manuscript.
