# Supplementary material for: A new FRDA mouse model [Fxnnull:YG8s(GAA) > 800] with more than 800 GAA repeats
Source: Front Neurosci. 2023 Jan 26;17:930422. doi: 10.3389/fnins.2023.930422 (PMC9909538; doi:10.3389/fnins.2023.930422)
Supplement: Supplementary file 1 [file Presentation_1.PPTX]

## Slide 1
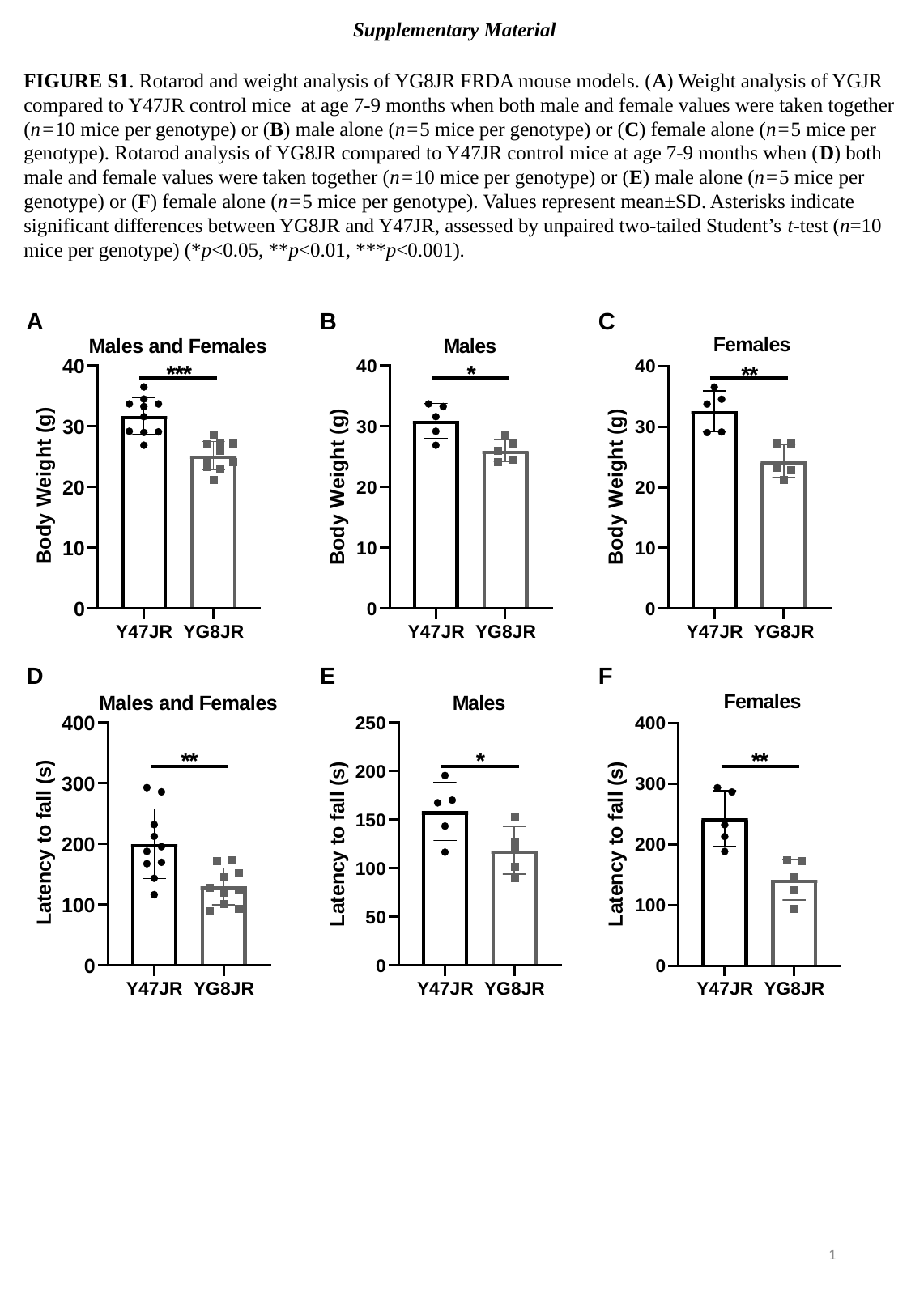

Supplementary Material
FIGURE S1. Rotarod and weight analysis of YG8JR FRDA mouse models. (A) Weight analysis of YGJR compared to Y47JR control mice at age 7-9 months when both male and female values were taken together (n = 10 mice per genotype) or (B) male alone (n = 5 mice per genotype) or (C) female alone (n = 5 mice per genotype). Rotarod analysis of YG8JR compared to Y47JR control mice at age 7-9 months when (D) both male and female values were taken together (n = 10 mice per genotype) or (E) male alone (n = 5 mice per genotype) or (F) female alone (n = 5 mice per genotype). Values represent mean±SD. Asterisks indicate significant differences between YG8JR and Y47JR, assessed by unpaired two-tailed Student’s t-test (n=10 mice per genotype) (*p<0.05, **p<0.01, ***p<0.001).
A
B
C
D
E
F
1

## Slide 2
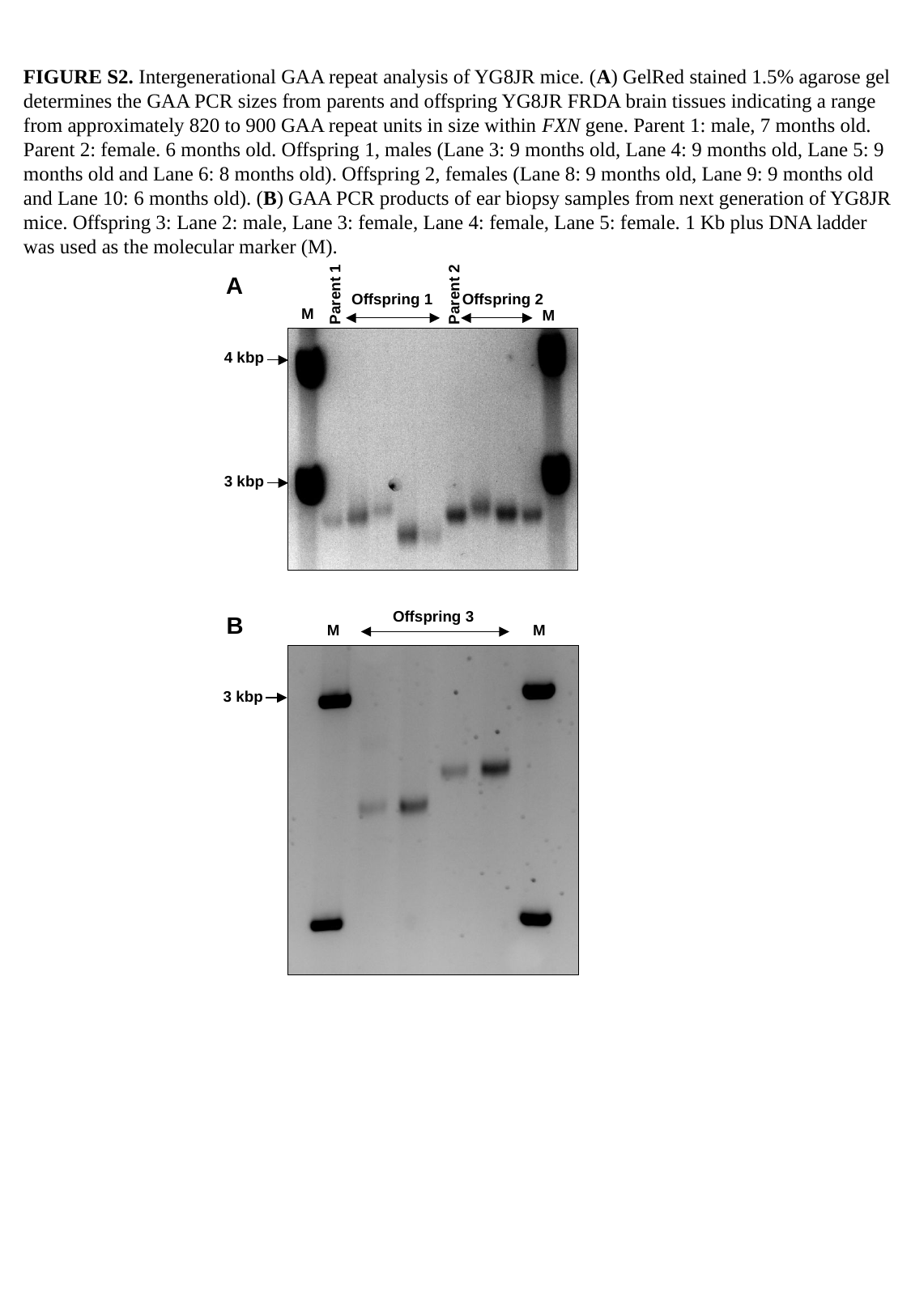

FIGURE S2. Intergenerational GAA repeat analysis of YG8JR mice. (A) GelRed stained 1.5% agarose gel determines the GAA PCR sizes from parents and offspring YG8JR FRDA brain tissues indicating a range from approximately 820 to 900 GAA repeat units in size within FXN gene. Parent 1: male, 7 months old. Parent 2: female. 6 months old. Offspring 1, males (Lane 3: 9 months old, Lane 4: 9 months old, Lane 5: 9 months old and Lane 6: 8 months old). Offspring 2, females (Lane 8: 9 months old, Lane 9: 9 months old and Lane 10: 6 months old). (B) GAA PCR products of ear biopsy samples from next generation of YG8JR mice. Offspring 3: Lane 2: male, Lane 3: female, Lane 4: female, Lane 5: female. 1 Kb plus DNA ladder was used as the molecular marker (M).
Parent 1
Parent 2
Offspring 1
Offspring 2
M
M
4 kbp
3 kbp
A
Offspring 3
B
M
M
3 kbp
2

## Slide 3
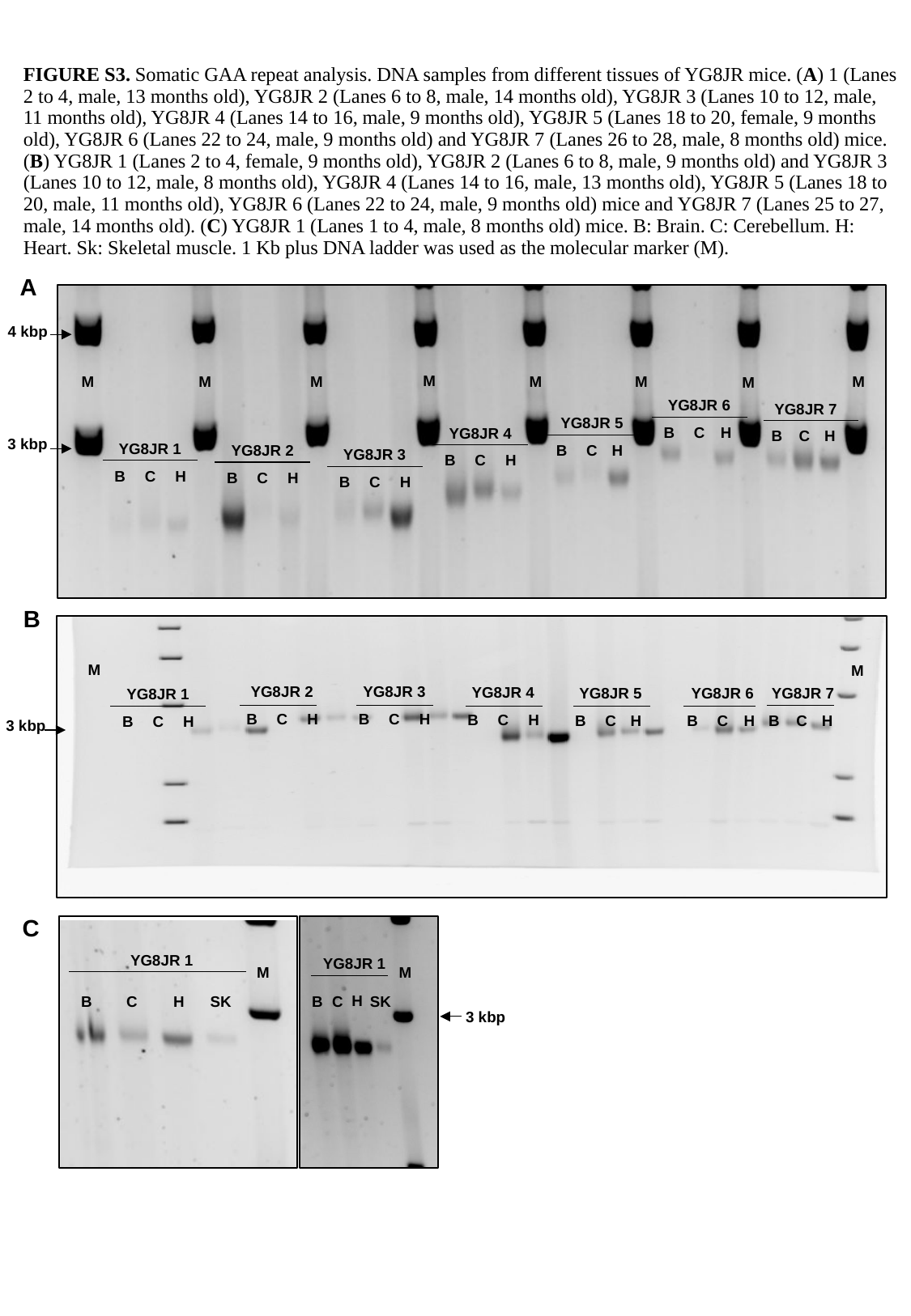

FIGURE S3. Somatic GAA repeat analysis. DNA samples from different tissues of YG8JR mice. (A) 1 (Lanes 2 to 4, male, 13 months old), YG8JR 2 (Lanes 6 to 8, male, 14 months old), YG8JR 3 (Lanes 10 to 12, male, 11 months old), YG8JR 4 (Lanes 14 to 16, male, 9 months old), YG8JR 5 (Lanes 18 to 20, female, 9 months old), YG8JR 6 (Lanes 22 to 24, male, 9 months old) and YG8JR 7 (Lanes 26 to 28, male, 8 months old) mice. (B) YG8JR 1 (Lanes 2 to 4, female, 9 months old), YG8JR 2 (Lanes 6 to 8, male, 9 months old) and YG8JR 3 (Lanes 10 to 12, male, 8 months old), YG8JR 4 (Lanes 14 to 16, male, 13 months old), YG8JR 5 (Lanes 18 to 20, male, 11 months old), YG8JR 6 (Lanes 22 to 24, male, 9 months old) mice and YG8JR 7 (Lanes 25 to 27, male, 14 months old). (C) YG8JR 1 (Lanes 1 to 4, male, 8 months old) mice. B: Brain. C: Cerebellum. H: Heart. Sk: Skeletal muscle. 1 Kb plus DNA ladder was used as the molecular marker (M).
A
4 kbp
3 kbp
M
M
M
M
M
M
M
M
YG8JR 6
B
C
H
YG8JR 7
B
C
H
YG8JR 5
B
C
H
YG8JR 4
B
C
H
YG8JR 1
B
C
H
YG8JR 2
B
C
H
YG8JR 3
B
C
H
B
M
M
YG8JR 3
B
C
H
YG8JR 2
B
C
H
YG8JR 4
B
C
H
YG8JR 5
B
C
H
YG8JR 7
B
C
H
YG8JR 6
B
C
H
YG8JR 1
B
C
H
3 kbp
C
YG8JR 1
C
H
SK
B
YG8JR 1
M
M
H
B
C
SK
3 kbp

## Slide 4
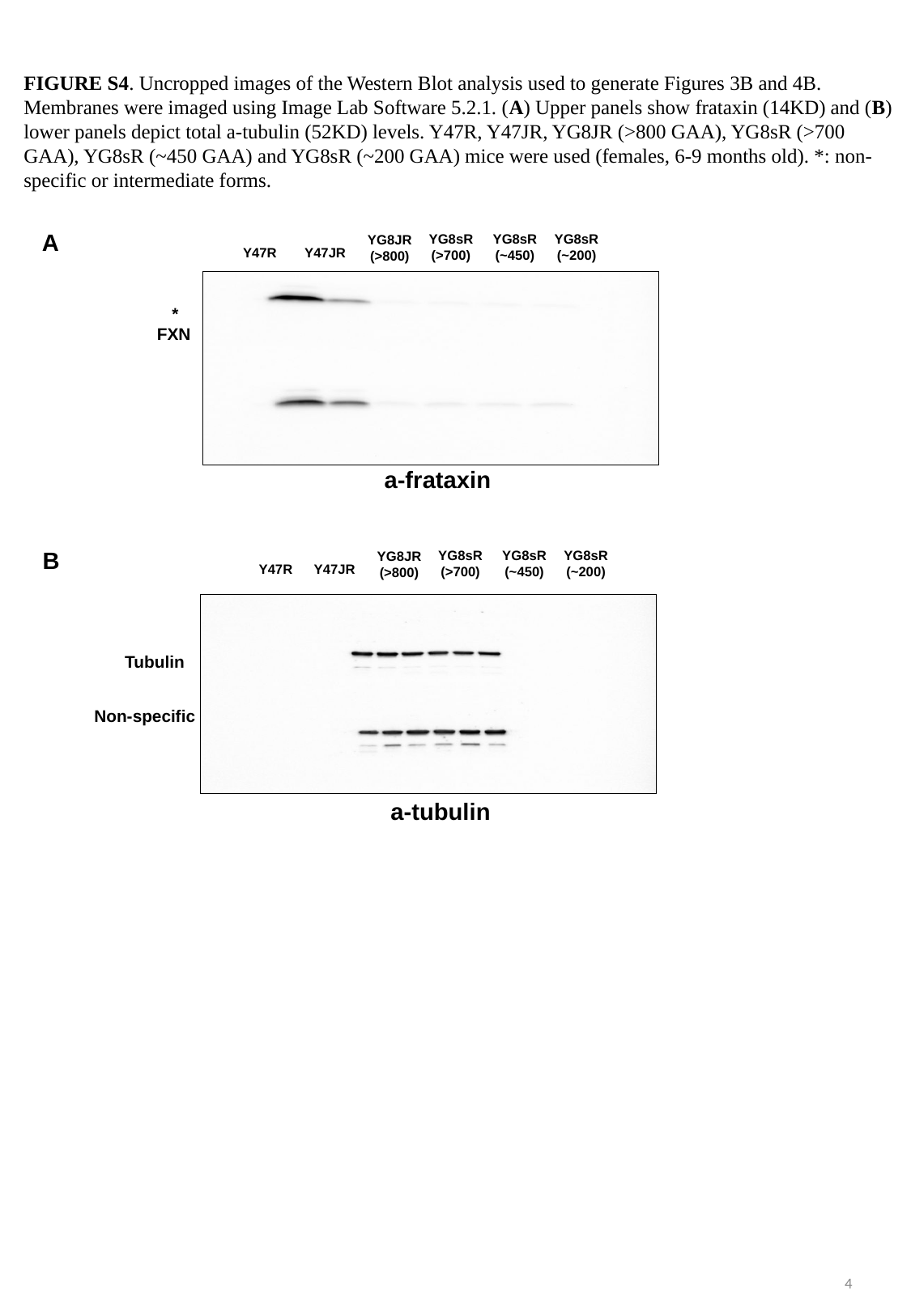

FIGURE S4. Uncropped images of the Western Blot analysis used to generate Figures 3B and 4B. Membranes were imaged using Image Lab Software 5.2.1. (A) Upper panels show frataxin (14KD) and (B) lower panels depict total a-tubulin (52KD) levels. Y47R, Y47JR, YG8JR (>800 GAA), YG8sR (>700 GAA), YG8sR (~450 GAA) and YG8sR (~200 GAA) mice were used (females, 6-9 months old). *: non-specific or intermediate forms.
A
YG8sR (~200)
YG8sR (~450)
YG8sR (>700)
YG8JR
(>800)
Y47R
Y47JR
*
FXN
a-frataxin
B
YG8sR (~200)
YG8sR (~450)
YG8sR (>700)
YG8JR
(>800)
Y47R
Y47JR
Tubulin
Non-specific
a-tubulin
4

## Slide 5
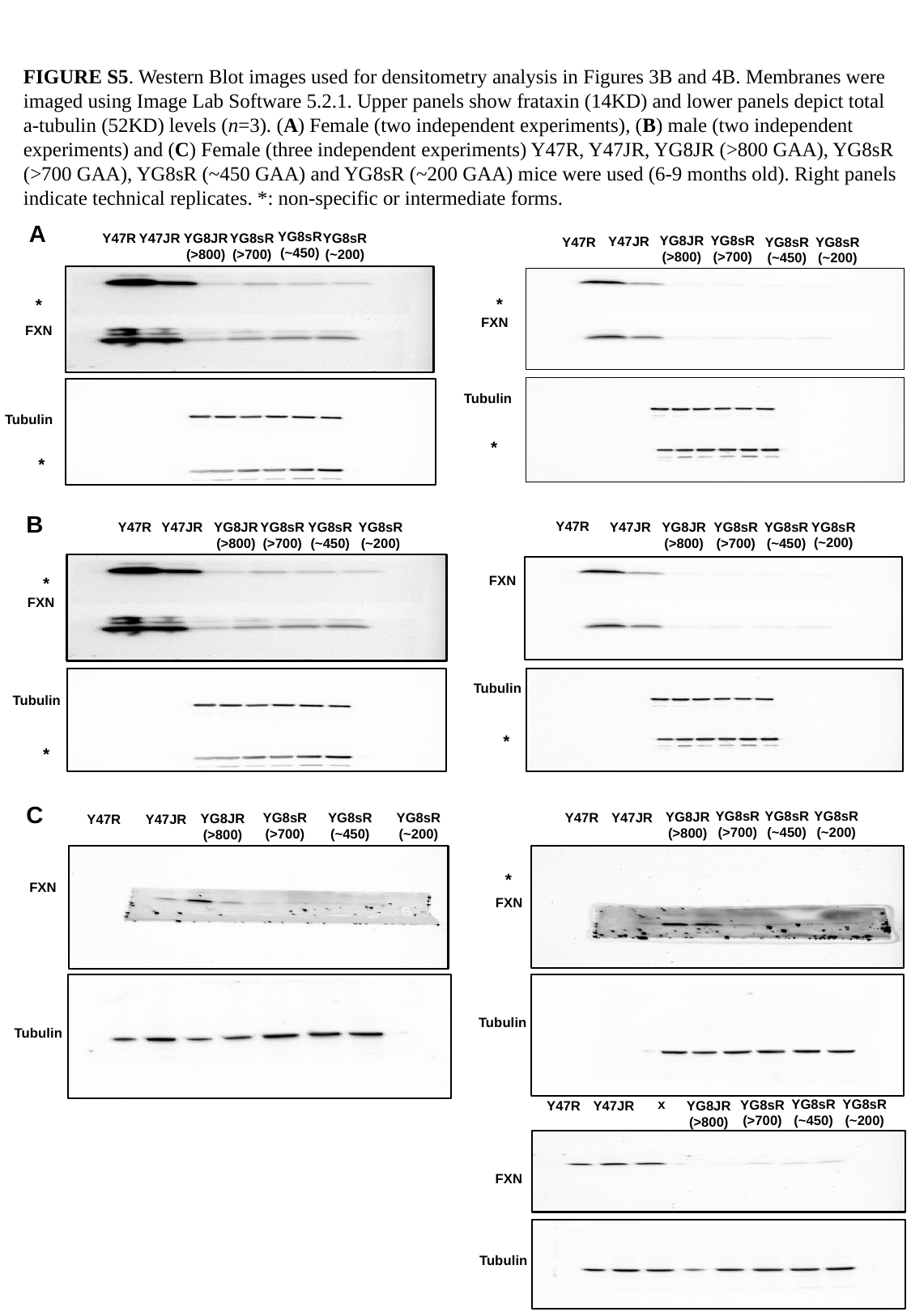

FIGURE S5. Western Blot images used for densitometry analysis in Figures 3B and 4B. Membranes were imaged using Image Lab Software 5.2.1. Upper panels show frataxin (14KD) and lower panels depict total a-tubulin (52KD) levels (n=3). (A) Female (two independent experiments), (B) male (two independent experiments) and (C) Female (three independent experiments) Y47R, Y47JR, YG8JR (>800 GAA), YG8sR (>700 GAA), YG8sR (~450 GAA) and YG8sR (~200 GAA) mice were used (6-9 months old). Right panels indicate technical replicates. *: non-specific or intermediate forms.
A
YG8sR (~450)
Y47JR
YG8JR
(>800)
Y47R
YG8sR (>700)
YG8sR (~200)
*
FXN
*
YG8sR (>700)
YG8JR
(>800)
Y47JR
Y47R
YG8sR (~450)
YG8sR (~200)
*
FXN
Tubulin
*
Tubulin
B
Y47R
Y47JR
YG8JR
(>800)
YG8sR (>700)
YG8sR (~200)
YG8sR (~450)
FXN
Tubulin
*
Y47JR
YG8JR
(>800)
Y47R
YG8sR (>700)
YG8sR (~200)
YG8sR (~450)
*
Tubulin
*
FXN
C
YG8sR (~200)
YG8sR (~450)
YG8sR (>700)
YG8JR
(>800)
Y47R
Y47JR
*
FXN
Tubulin
YG8sR (~200)
YG8sR (~450)
YG8sR (>700)
YG8JR
(>800)
Y47R
Y47JR
FXN
Tubulin
YG8sR (~200)
YG8sR (~450)
YG8sR (>700)
YG8JR
(>800)
Y47R
Y47JR
FXN
Tubulin
x
