# Supplementary material for: A new FRDA mouse model [Fxnnull:YG8s(GAA) > 800] with more than 800 GAA repeats
Source: Front Neurosci. 2023 Jan 26;17:930422. doi: 10.3389/fnins.2023.930422 (PMC9909538; doi:10.3389/fnins.2023.930422)
Supplement: Supplementary file 2 [file Presentation_2.PPTX]

## Slide 1
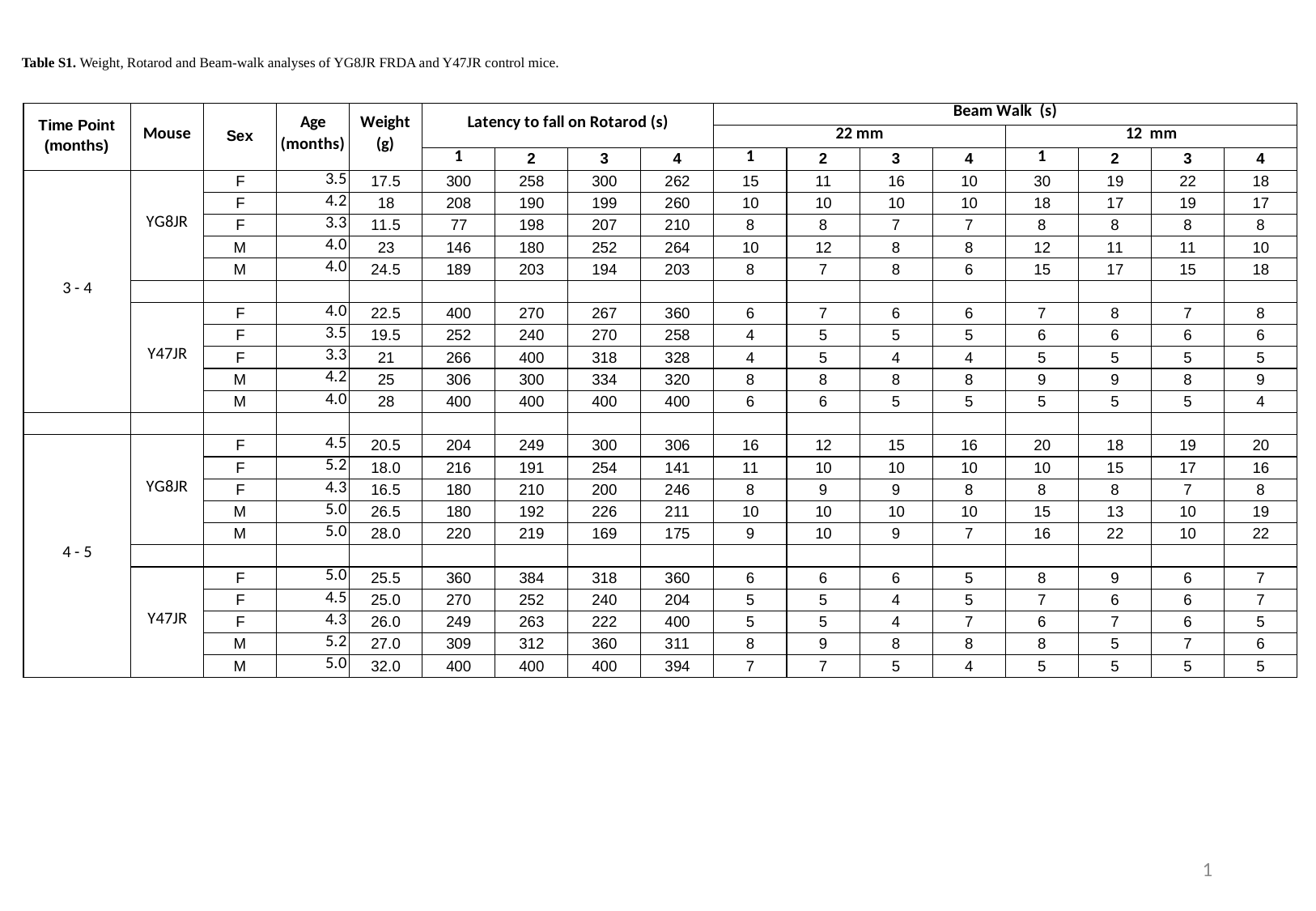

Table S1. Weight, Rotarod and Beam-walk analyses of YG8JR FRDA and Y47JR control mice.
1

## Slide 2
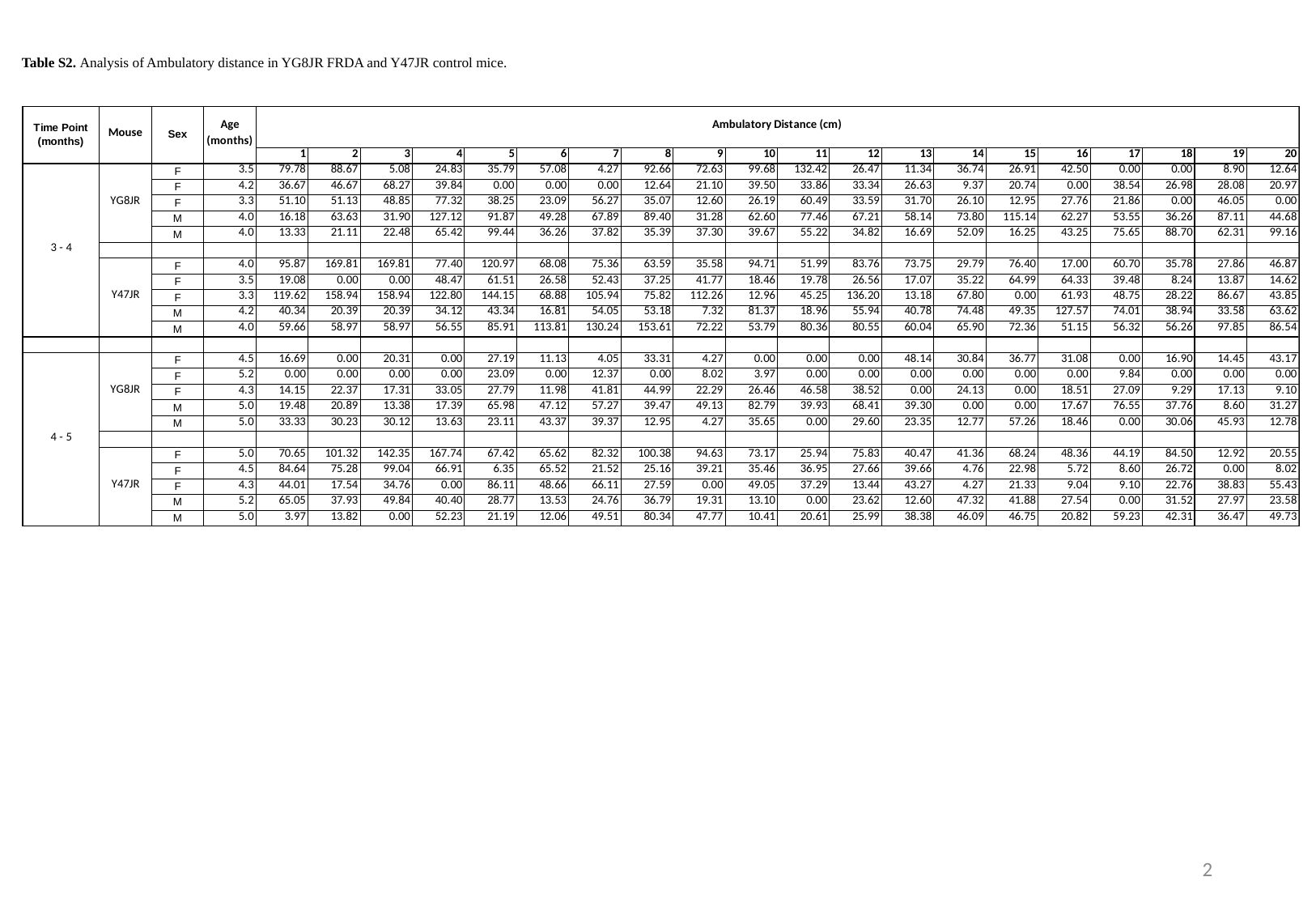

Table S2. Analysis of Ambulatory distance in YG8JR FRDA and Y47JR control mice.
2

## Slide 3
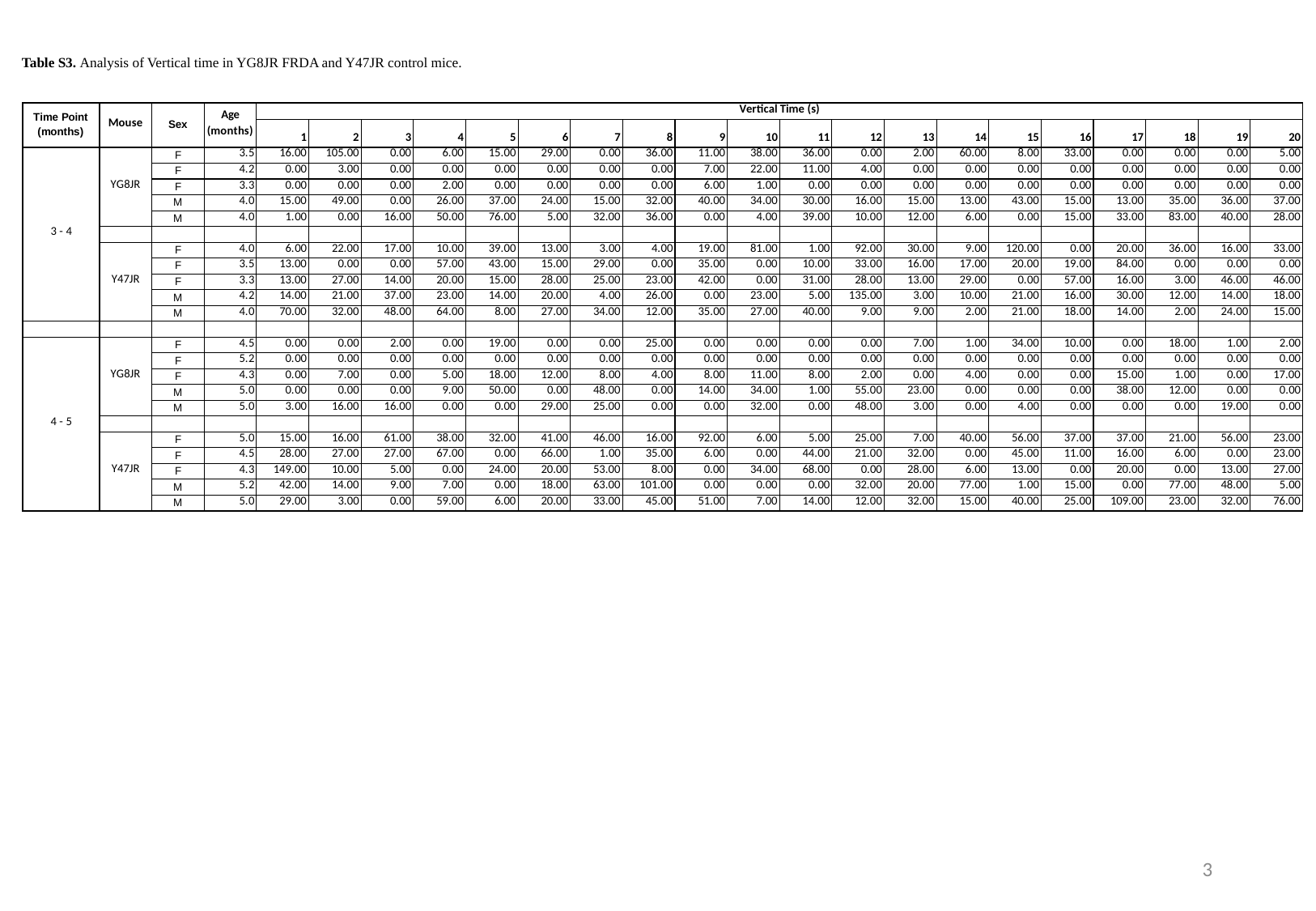

Table S3. Analysis of Vertical time in YG8JR FRDA and Y47JR control mice.
3

## Slide 4
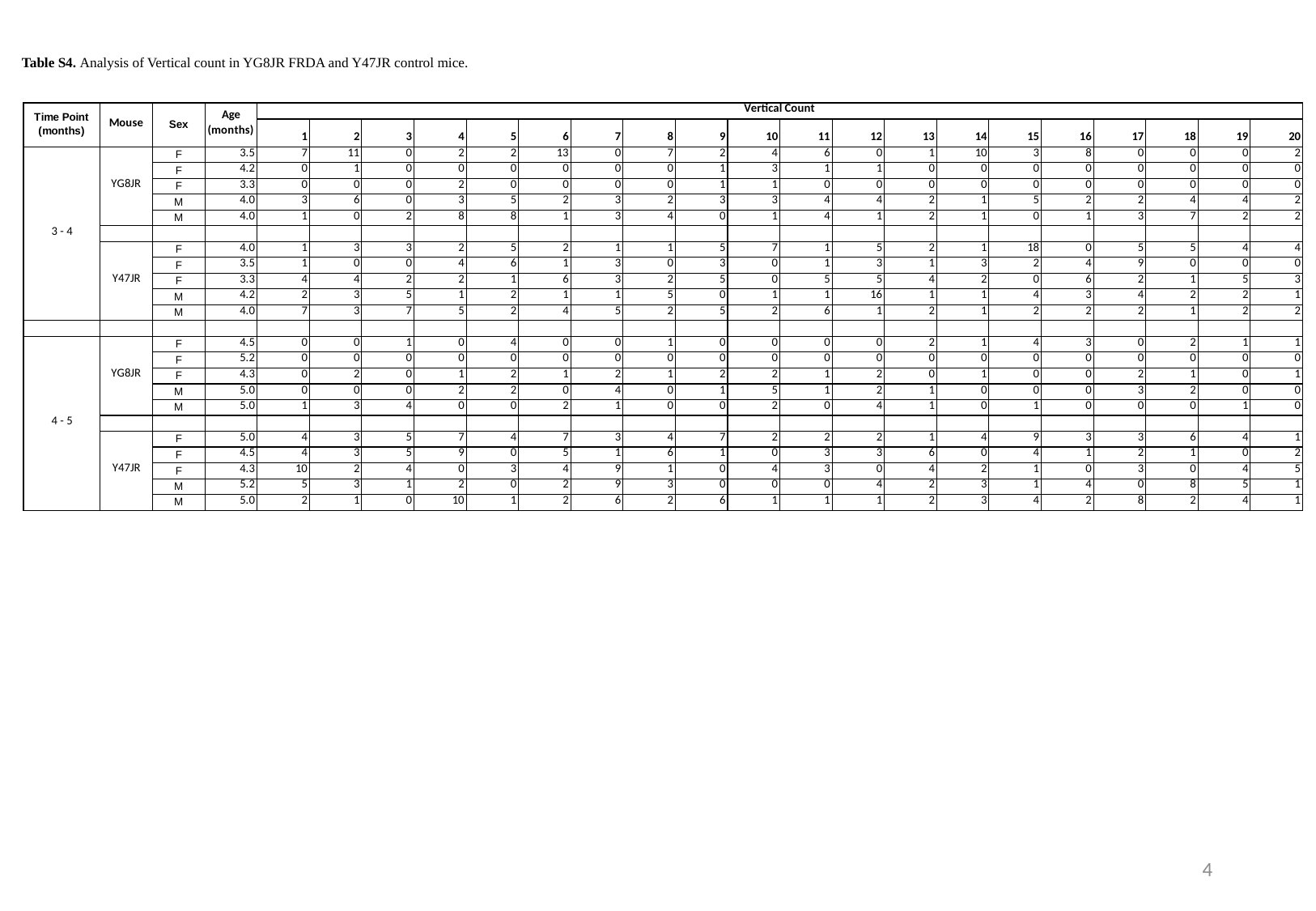

Table S4. Analysis of Vertical count in YG8JR FRDA and Y47JR control mice.
4

## Slide 5
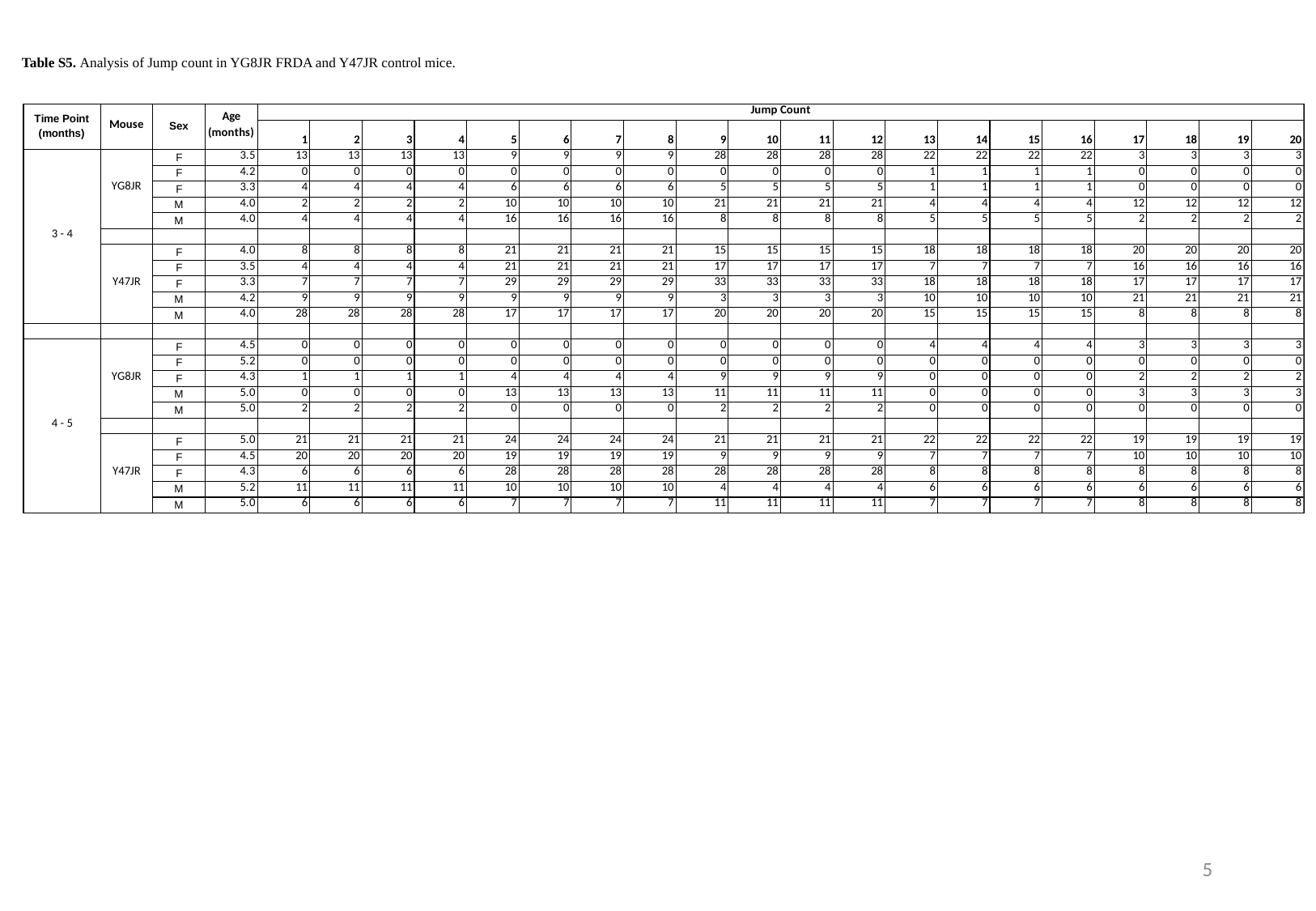

Table S5. Analysis of Jump count in YG8JR FRDA and Y47JR control mice.
5

## Slide 6
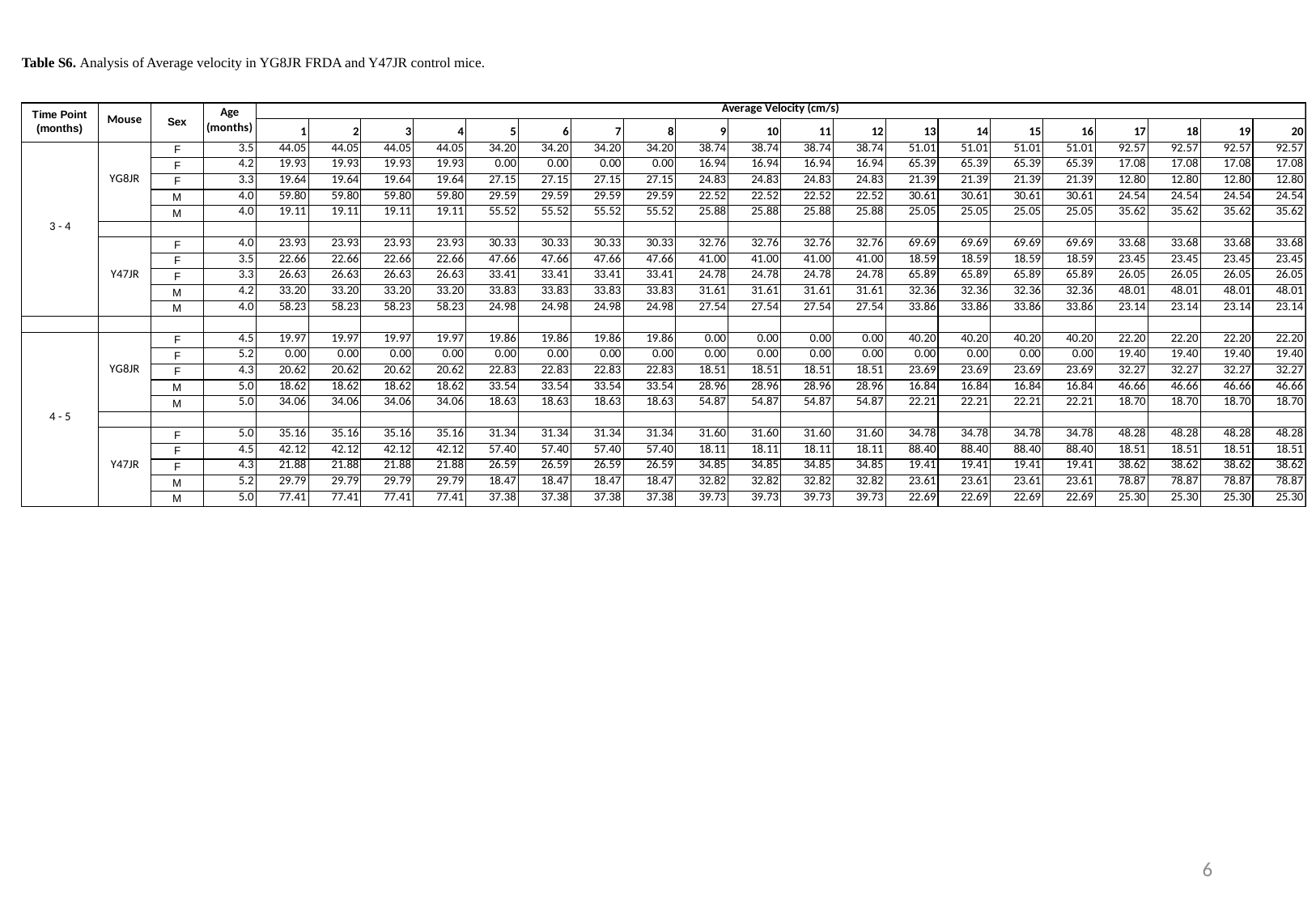

Table S6. Analysis of Average velocity in YG8JR FRDA and Y47JR control mice.
6
